# Diagnostic Value of Serum Pepsinogen Levels for Screening Gastric Cancer and Atrophic Gastritis in Asymptomatic Individuals: A Cross-Sectional Study

**DOI:** 10.3389/fonc.2021.652574

**Published:** 2021-08-24

**Authors:** Yuling Tong, Hongguang Wang, Yi Zhao, Xueqiang He, Hongwei Xu, Hong Li, Ping Shuai, Lirong Gong, Hongbo Wu, Hongzhi Xu, Yinhu Luo, Dong Wang, Shizhu Liu, Zhenya Song

**Affiliations:** ^1^Department of General Practice/Health Management Center, The Second Affiliated Hospital of Zhejiang University, School of Medicine, Hangzhou, China; ^2^Department of Gastroenterology, Jilin City People’s Hospital, Jilin, China; ^3^Department of Gastroenterology, No. 924 Hospital of the People’s Liberation Army of China, Guilin, China; ^4^Department of Gastroenterology, Kunshan Hospital of Traditional Chinese Medicine, Kunshan, China; ^5^Department of Health Medicine, Chinese People’s Liberation Army (PLA) General Hospital, Beijing, China; ^6^Health Management Center, Sichuan Provincial People’s Hospital, University of Electronic Science and Technology of China, Chengdu, China; ^7^Department of Gastroenterology, The First Hospital Affiliated to AMU (Southwest Hospital), Chongqing, China; ^8^Department of Gastroenterology, Zhongshan Hospital Affiliated to Xiamen University, Xiamen, China; ^9^Department of Gastroenterology, Jingzhou Hospital of Traditional Chinese Medicine, Jingzhou, China; ^10^Department of Gastroenterology, Shanghai Changhai Hospital, Shanghai, China

**Keywords:** pepsinogens, gastric cancer, precancerous lesions, *Helicobacter pylori*, screening, diagnostic value

## Abstract

**Background:**

Pepsinogens (PGs) can be used for gastric cancer (GC) screening, but the cutoff levels vary among studies, and PG levels are influenced by numerous factors. The aim of this article is to examine the diagnostic value of PG levels and Helicobacter pylori (Hp) status for GC and atrophic gastritis screening in asymptomatic individuals undergoing health checkup in China.

**Patients and Methods:**

This was a multicenter cross-sectional study of subjects who underwent health checkup from 10/2016 to 10/2018 at nine International Healthcare Centers in China. All participants underwent gastroscopy and pathological examination, serum PG, ^13^C-urea breath test, and/or *Hp* serological current infection marker rapid test, all on the same day. PG-related parameters were analyzed in different *Hp* subgroups and regions.

**Results:**

The patients were grouped as non-atrophic (NAG, n = 1,590), mild to moderate atrophic (MAG, n = 273), severe atrophic (SAG, n = 49), and GC (n = 10). The serum PG levels in these groups decreased with increasing pathological severity. In the same pathological groups, PGI and PGII levels were higher in the *Hp*-positive subgroup, while PGR (PGI/PGII ratio) was lower (P < 0.05). The best cutoff values for atrophy diagnosis were PGI ≤73.1 ng/ml and PGR ≤9.8, for severe atrophy were PGI ≤63.9 ng/ml and PGR ≤9.09, and for GC was PGR ≤4.7 (all P < 0.05 and area under the curve >0.7). The cutoff points varied with *Hp* status and China regions.

**Conclusion:**

Serum PG levels might be used for the screening of gastric atrophic gastritis lesions. The results suggest that different cutoff values should possibly be used in different *Hp* status groups and geographical regions, but it will have to be validated in future studies. Future studies should also examine the value of PG levels for GC detection.

## Introduction

Gastric cancers (GCs) are tumors of the stomach, including non-cardia and subcardia carcinomas (Siewert type III), with the center starting 2–5 cm below the esophagogastric junction (1, 2). GC affected approximately 1,033,701 individuals globally in 2018 (3), and its incidence is highest in Eastern Asia, Eastern Europe, and South America (2, 4). Men are twice as likely to be affected as women (2). The direct cause of GC is unclear, but *Helicobacter pylori* (*Hp*) infection and hereditary cancer predisposition syndromes may play a role (5, 6). Patients often present with non-specific symptoms, which may include anorexia, weight loss, abdominal pain, dyspepsia, vomiting, and early satiety (2, 4). In China, the 5-year survival rate of patients with GC is 27.4% (7, 8).

The progression from chronic non-atrophic gastritis, *via* atrophic gastritis (AG) and intestinal metaplasia (IM), to dysplasia, termed Correa’s cascade (9), is widely considered a common evolution path of the intestinal type of non-cardia GC. AG is the turning point and represents a precancerous lesion. The annual incidence rates of gastric cancer were found to be 0.1, 0.25, 0.6, and 6% in patients with AG, IM, mild-to-moderate dysplasia, and severe dysplasia within 5 years after diagnosis (10). Effective screening and managing of this patient group could reduce the incidence of GC and improve the early detection rate. Gastroscopy followed by pathology is the gold standard for the diagnoses of GC and precancerous lesions, but the large target population of GC endoscopic screening and the lack of high-quality medical resources are major impediments to its implementation (11, 12). In addition, patients with early GC and pre-gastric cancer usually have no specific symptoms (11, 12).

Pepsinogen (PG) is a zymogen abundantly secreted by gastric mucosal cells that is converted into pepsin by the acidic pH in the gastric lumen (13). There are five different groups of PGs, grouped according to their primary structure: PGI, PGII, PGB, prochymosin, and PGF (13). Serum PG amounts can reflect the morphology and function of the gastric mucosa. Indeed, PGI is produced by the adenosine cells of the gastric fundus, and low PGI levels correlate with gastric precancerous lesions and GC (14–16). PGII is more correlated with gastric mucosal lesions compared with those of the gastric antrum mucosa; in addition, PGII is related to gastric duct atrophy, intestinal metaplasia or pseudopyloric gland metaplasia, and dysplasia (17, 18). Interestingly, dysregulated PG expression and a progressive decrease of the PGI/PGII ratio (PGR) are associated with the progression from normal gastric mucosa to precancerous lesions to GC (15, 19). Additionally, PGR plays a critical role in the detection of AG cases (20). *Hp* has been identified as a carcinogen by the World Health Organization (WHO) (21, 22). The incidence of non-cardiac GC in *Hp*-positive individuals is 2.97 times that of negative ones (23). A study in Taiwan found that compared with *Hp*-positive patients, *Hp*-negative individuals are more likely to develop proximal GC and tend to be younger with more diffuse lesions and worse prognosis (24).

The combination of *Hp* and PG has been used to evaluate the risk factors for GC, which is probably more suitable for large-scale screening than endoscopy (21). Nevertheless, its efficacy remains unsure, and its cutoff value varies among studies; meanwhile, serum PG levels are affected by race, region, age, gender, height, weight, body surface area, smoking, and alcohol, among others (25). Previous studies showed that serum PG levels and its relationship with GC in China are significantly different from those found in Japan and South Korea (20, 26, 27). There is a high incidence of *Hp* infection in China, and many studies have shown that PGI and PGII levels increase and PGR decreases after *Hp* infection. A previous single-center study by our group (20) discussed the classification of PG cutoff values according to *Hp* infection.

Therefore, the aim of this multicenter cross-sectional study was to examine PG levels and *Hp* status in asymptomatic individuals undergoing health checkup in order to assess the diagnostic value of serum PG levels for GC and AG.

## Materials and Methods

### Participants

This was a multicenter cross-sectional study of consecutive subjects who underwent regular health checkup from October 2016 to October 2018 at nine International Healthcare Centers in different regions of China, including Southern China (No. 924 Hospital of the People’s Liberation Army of China), Eastern China (the Second Affiliated Hospital of Zhejiang University, College of Medicine, Zhongshan Hospital affiliated to Xiamen University, and Traditional Chinese Medicine Hospital of Kunshan), Southwest China [Sichuan Provincial People’s Hospital and the First Hospital affiliated to AMU (Southwest Hospital)], Northeast China (Jinlin People’s Hospital), and Central/Northern China (Chinese PLA General Hospital and Jingzhou Hospital of Traditional Chinese Medicine). All procedures were performed in accordance with the ethical standards of the responsible committee on human experimentation (institutional and national) and the Helsinki Declaration of 1964 and later versions. Informed consent was obtained from all patients prior to enrolment. The study was approved and authorized by the ethics committees of various participating hospitals (approval #2015-082 at the Second Affiliated Hospital of Zhejiang University College of Medicine, the leading site).

Inclusion criteria were as follows (1): intention to undergo gastroscopy during health checkup examination and (2) 25–75 years of age. Exclusion criteria were the following: (1) a history of gastric ulcer, gastric polyp, or GC; (2) a history of gastrectomy; (3) treatment with a proton pump inhibitor in the last month; (4) contraindications to gastroscopy; (5) a history of *Hp* eradication; (6) a history of abdominal pain, abdominal distention, belching, acid reflux, nausea, and other digestive tract symptoms within 1 month; or (7) incomplete data.

### Questionnaire Survey

A self-reported questionnaire was used in the present study. It included baseline information (age, sex, and nationality), living style [smoking (>1 cigarette daily for more than 1 year; the number of cigarettes and duration of smoking were asked for smokers), alcohol consumption (any type of alcohol more than once weekly for more than 1 year; alcohol types and consumption frequency were asked for drinkers)], eating habits [high-salt diet (salt >10 g/day), green vegetables and fresh fruits [>three times per week)], and family history of GC among first-degree relatives (online **Supplementary File 1**).

### Tests

The participants underwent gastroscopy and pathological examination of the biopsies, serum PG test, ^13^C-urea breath test (Shenzhen Zhonghe Headway Bio-Sci & Tech Co., Ltd., China), and/or *Hp* serological current infection marker rapid test (MP Biomedicals, Santa Ana, CA, USA), all on the same day. All tests were performed according to the manufacturers’ instructions.

*Hp* infection was determined based on the ^13^C-urea breath test, *Hp* serological current infection marker rapid test (28), and pathological screening. Patients showing positive results for any of these three tests were considered to be *Hp*-positive. If all tests were negative, the patient was considered to be *Hp*-negative.

Fasting blood (5 ml) was collected from each subject and centrifuged for 10 min at ≥10,000 g. Serum PG levels were assayed by the chemiluminescent microparticle immunoassay method with the Abbott ARCHITECT Pepsinogen I and II Reagent Kit (Abbott Laboratories Inc., Chicago, IL, USA).

Gastroscopy was performed by the double-blind method. Two biopsies were performed at the small curvatures of the gastric antrum and body, respectively. Additional biopsies were taken from the mucosal abnormalities. The biopsies were scored semiquantitatively by two pathologists with >10 years of experience, according to the updated Sydney classification system (29) and the OLGA (Operative Link on Gastritis Assessment) method, which combine the degree and range of gastric mucosa atrophy/intestinal metaplasia, which are internationally accepted and applied in the screening of GC and AG (30). The OLGA-0 group was defined as normal (NAG), the combined OLGA-I and OLGA-II groups as mild-to-moderate atrophy (MAG), and the combined OLGA-III and OLGA-IV groups as severe atrophy (SAG). Therefore, based on pathological data, the patients were divided into four groups, including the non-atrophy (NAG), mild-to-moderate atrophy (MAG), severe atrophy (SAG), and GC groups. The last three groups were further combined into the AG and GC (AG/GC) group. In case of disagreement, the two pathologists discussed the data until consensus was reached.

### Quality Control

In the initial stage of the study, all participants underwent a unified training, including how to complete the questionnaire, how to perform the endoscopy, the training of the endoscopists, and the training of the pathologists. All pathological examinations were performed by two experts. Any inconsistencies were solved by discussion. The final conclusion was used in the database; therefore, no coefficients of variation could be calculated.

### Statistical Analysis

Statistical analysis was performed with SPSS 20 (IBM Corp., Armonk, NY, USA). Continuous data were tested for normal distribution by the Kolmogorov-Smirnov test. Those with normal distribution were expressed as mean ± standard deviation (SD) and compared by ANOVA with *post hoc* Scheffe’s test; data with skewed distribution were presented as median (interquartile ranges) (IQR). Categorical data were presented as frequency and percentage and analyzed by the chi-square test and Bonferroni *post hoc* test. A binary logistic regression model was used to determine odds ratios of potential risk factors for GC and AG. The receiver operating characteristics (ROC) curve method was used to estimate the cutoff points for PGs, according to the following steps. First, the original concentrations in the NAG population (derivation cohort) were logarithmically transformed and divided equally into 20 parts to obtain 20 cutoff values. Then, the prevalence of AG/GC for each of the 20 cutoffs was calculated, and cutoff categories with similar prevalence rates were combined for two final PG categories. Finally, the antilog values of the cutoffs of the combined categories were calculated and considered the cutoff values for PGs. An area under the curve (AUC) >0.7 was considered valuable. P < 0.05 was considered statistically significant.

## Results

### Basic Information

A total of 2,256 subjects were included in the study. Totally, 15 cancers were found, including 14 GCs (including six cases of early GC) and one pharyngeal cancer. There were 37 intraepithelial neoplasia (1.6%), 172 gastric polyp (7.6%), 87 gastric ulcer (3.9%), 58 duodenal ulcer (2.6%), 17 submucosal gastric tumor (0.8%), 326 AG (14.5%), and 391 intestinal metaplasia (17.3%) cases. In addition, one esophageal ulcer and one esophageal polyp cases were found.

Totally. 316 patients were excluded due to incomplete data, four due to a history of gastrectomy, five due to proton pump inhibitor use in the recent 1 month, and nine due to a history of gastric ulcer or gastric polyp. Finally, 1,922 patients were included in the final analysis. The participants were 52.3 ± 9.8 years old. The male to female ratio was 1.2:1 (1,065/857). There were 1,590 participants in the NAG group and 332 in the AG/GC group, including 273 in the MAG group, 49 in the SAG group, and 10 in the GC group. Compared with the NAG group, mean age, fruit intake, vegetable intake, smoking rate, and the infection rate of *Hp* in the AG/GC group were significantly different (P < 0.05) (**Table 1**).

**Table 1:** The baseline characteristics of 1,922 subjects involved in the study, with univariate and multivariable analyses.

Characteristics	Total (n = 1,922)	NAG group (n = 1,590)	AG/GC group (n = 332)				P value^§^	Adjusted OR (95% CI)^†^	P value
			Total (n = 332)	MAG group (n = 273)	SAG group (n = 49)	GC group (n = 10)			
Sex							0.265		
Male, n (%)	1,065	866 (54.5)	199 (59.9)	166 (60.8)	28 (57.1)	5 (50.0)			
Female, n (%)	857	724 (45.5)	133 (40.1)	107 (29.2)	21 (42.9)	5 (50.0)			
Age (years)							<0.001		
<50	753	653 (41.1)	100 (30.1)	87 (31.9)	11 (22.5)	2 (20.0)		Reference	
50–59	720	573 (36.0)	147 (44.3)	121 (44.3)	23 (46.9)	3 (30.0)		1.76 (1.31–2.34)	<0.001
60–69	367	302 (19.0)	65 (19.6)	50 (18.3)	12 (24.5)	3 (30.0)		1.75 (1.22–2.52)	0.002
>70	82	62 (3.9)	20 (6.0)	15 (5.5)	3 (6.1)	2 (20.0)		2.93 (1.65–5.21)	<0.001
mean (SD)	52.3 (9.8)	51.9 (9.9)	54.3 (9.3)	53.8 (8.9)	55.5 (9.7)	59.8 (15.0)	0.001		
Nation							0.087		
Minority, n (%)	114	101 (6.4)	13 (3.9)	9 (3.3)	3 (6.1)	1 (10.0)			
Han nationality, n (%)	1,808	1,489 (93.6)	319 (96.1)	264 (96.7)	46 (93.9)	9 (90.0)			
Family history							0.414		
No, n (%)	1,655	1,382 (86.9)	283 (85.2)	232 (85.0)	44 (89.8)	7 (70.0)			
Yes, n (%)	257	208 (13.1)	49 (14.8)	41 (15.0)	5 (10.2)	3 (30.0)			
High-salt diet							0.061		
No, n (%)	1,645	1,350 (84.9)	295 (88.9)	242 (88.6)	46 (93.9)	7 (70.0)			
Yes, n (%)	277	240 (15.1)	37 (11.1)	31 (11.4)	3 (6.1)	3 (30.0)			
Fruits							<0.001		
Occasional	662	585 (36.8)	77 (23.2)	66 (24.2)	8 (16.3)	3 (30.0)		1.90 (1.38–2.63)	<0.001
Regular	1,260	1,005 (63.2)	255 (76.8)	207 (75.8)	41 (83.7)	7 (70.0)		Reference	
Vegetables							0.011		
Occasional	198	174 (10.9)	24 (7.2)	20 (7.3)	1 (2.0)	3 (30.0)			
Regular	1,724	1,416 (89.1)	308 (92.8)	253 (92.7)	48 (98.0)	7 (70.0)			
Milk							0.176		
Occasional	1,475	1,217 (76.5)	258 (77.7)	209 (76.6)	43 (87.8)	6 (60.0)			
Regular	447	373 (23.5)	74 (22.3)	64 (23.4)	6 (12.2)	4 (40.0)			
Smoking							0.008		
No, n (%)	1,396	1,180 (74.2)	216 (65.1)	177 (64.8)	33 (63.7)	6 (60.0)		Reference	
Yes, n (%)	526	410 (25.8)	116 (34.9)	96 (35.2)	16 (32.7)	4 (40.0)		1.80 (1.33–2.47)	<0.001
Drinking							0.919		
No, n (%)	1,336 (69.5)	1,106 (69.6)	230 (69.3)	185 (67.8)	37 (75.5)	8 (80.0)			
Yes, n (%)	586 (30.5)	484 (30.4)	102 (30.7)	88 (32.3)	12 (24.5)	2 (20.0)			
*Hp* infection							<0.001		
Negative, n (%)	1,165	1,007 (63.3)	158 (47.6)	136 (49.8)	17 (34.7)	5 (50.0)		Reference	
Positive, n (%)	757	583 (36.7)	174 (52.4)	137 (50.2)	32 (65.3)	5 (50.0)		2.00 (1.54–2.59)	<0.001
PGI (ng/ml)							<0.001		
>151.5	388	339	49	43	5	1		Reference	
41.9–151.5	1,304	1,079	225	184	35	6		3.07 (1.80–5.22)	<0.001
≤41.8	230	172	58	46	9	3		1.56 (1.05–2.31)	0.027
Mean (SD)	86.9 (82.3)	90.4 (85.2)	67.8 (71.0)	69.5 (76.1)^*^	60.8 (53.8)^*^	64.9 (77.5)	0.001		
PGII (ng/ml)							0.689		
>22.0	208	168	40	29	8	3			
4.2–22.0	1,480	1,226	254	209	38	7			
≤4.1	234	196	38	35	3	0			
Mean (SD)	8.5 (8.8)	8.3 (8.6)	9.2 (9.7)	8.7 (9.4)	11.9 (12.3)^*^	11.1 (19.7)	0.246		
PGR							<0.001		
>5.49	1,580	1,332	248	215	29	4		Reference	
≤5.49	342	258	84	58	20	6		1.44 (1.03–2.00)	0.034
Mean (SD)	9.3 (8.6)	10.1 (9.3)	7.7 (5.2)	7.9 (5.4)^*#^	6.3 (4.5)^*#^	4.5 (10.2)^*#^	0.006		
Regions, n (%)									
Southern China	257	254 (16.0)	3 (1.0)	3 (1.1)	0 (0)	0 (0)			
Eastern China	781	574 (36.1)	207 (62.3)	165 (60.4)	38 (77.6)	4 (40.0)			
Southwest China	268	210 (13.2)	58 (17.5)	50 (18.3)	6 (12.2)	2 (20.0)			
Northeast China	386	332 (20.9)	54 (16.3)	47 (17.2)	5 (10.2)	2 (20.0)			
Central/Northern China	230	220 (13.8)	10 (3)	8 (2.9)	0 (0)	2 (20.0)			

Data are presented as n (%) for categorical variables, mean (SD) or median (range interquartile) for continuous variables. For variables about eating habits, two categories for frequency of consumption were provided, that is, occasional (<3 times/week) and regular (at least three times/week).

^§^P values refer to comparison between NAG and AG/GC groups in the univariate analysis.

^†^For variables not significant (p > 0.05) in the logistic regression model, multivariable data are not shown.

*P < 0.05 vs. the NAG group.

^#^P < 0.05 vs. the MAG group.

NAG, non-atrophic; MAG, mild-moderate atrophic; SAG, severe atrophic; GC, gastric cancer; PGI, pepsinogen I; PGII, pepsinogen II; PGR, PGI/PGII ratio; Hp, Helicobacter pylori.

### Risk Factors for AG/GC

According to the logistic regression model, age was one of the risk factors for AG/GC, especially individuals older than 70 (OR = 2.93). Other risk factors included occasional fruit intake (OR = 1.9), smoking (OR = 1.8), *Hp* infection (OR = 2.0), and PGR levels (OR = 1.44) (**Table 1**).

### Serum PG Differences Among the Pathological Groups

Serum PG levels were compared among the NAG, MAG, SAG, and GC groups. PGR values gradually decreased with increasing lesion grade. Compared with the NAG group, PGI levels and PGRs in the MAG and SAG groups were significantly lower (P < 0.05). Compared with the MAG group, the SAG group had significantly lower PGRs (P < 0.05). PGR levels in the GC group were significantly lower than those of the NAG and MAG groups (P < 0.05) (**Table 1**).

### Serum PG Differences According to the Hp Status

The groups were subdivided according to the *Hp* status. In the NAG group, PGI and PGII levels in the *Hp*-positive subgroup were higher than those of *Hp*-negative patients (P < 0.001 and P < 0.001), while PGRs were lower (P < 0.001). Similar changes were observed in the MAG group (P < 0.001, P < 0.001, and P = 0.009). In the SAG group, PGII levels in the *Hp*-positive subgroup were higher than those of the *Hp*-negative subgroup (P = 0.005), while PGRs were lower (P < 0.001) (**Table 2**).

**Table 2:** Differences in serum PG levels among the NAG, MAG, SAG, and GC group in subgroup analysis based on *Hp* infection.

	NAG group		MAG group		SAG group		GC group	
	*Hp*− (n = 1,007)	*Hp*+ (n = 583)	*Hp*− (n = 136)	*Hp*+ (n = 137)	*Hp*− (n = 17)	*Hp*+ (n = 32)	*Hp*− (n = 5)	Hp+ (n = 5)
PGI (ng/ml)	82.2 ± 78.7	101.7 ± 89.0^*^	55.8 ± 64.7^#^	81.2 ± 79.4^*#^	59.1 ± 41.6^#^	60.8 ± 57.1^#$^	43.5 ± 174.7	89.7 ± 67.0
PGII (ng/ml)	7.4 ± 6.6	10.8 ± 11.4^*^	7.1 ± 6.0	11.7 ± 10.2^*^	6.9 ± 6.2	15.3 ± 10.4^*^	7.5 ± 33.3	12.4 ± 13.2
PGR	10.7 ± 8.8	8.8 ± 9.9^*^	8.5 ± 4.7 ^#^	7.2 ± 6.0^*#^	8.9 ± 2.8^#^	4.5 ± 3.2^*#$^	4.7 ± 7.4	4.3 ± 11.9

^*^P < 0.05 vs. the Hp-negative group among the same pathology.

^#^P < 0.05 vs. the NAG group with the same Hp infection condition.

^$^P < 0.05 vs. the MAG group with the same Hp infection condition.

NAG, non-atrophic; MAG, mild-moderate atrophic; SAG, severe atrophic; GC, gastric cancer; PGI, pepsinogen I; PGII, pepsinogen II; PGR, PGI/PGII ratio; Hp, Helicobacter pylori.

In the *Hp*-positive population, PGI and PGR levels in the MAG and SAG groups were lower than those of the NAG group (P = 0.007 and P = 0.002; P < 0.001 and P < 0.001). Meanwhile, PGI and PGR levels in the SAG group were significantly lower than those of the MAG group (P = 0.026 and P < 0.001). In *Hp*-negative subjects, PGI and PGR levels in the MAG and SAG groups were lower than those of the NAG group (all P < 0.001) (**Table 2**).

### Serum PG Differences in Various Regions

The participants were subgrouped according to regions. In the NAG group, there were significant differences in PGI levels among the five Chinese regions assessed. There were also significant differences in PGII levels among the five regions, except for Central/Northern *vs.* Southern China. PGR levels in Southern China were higher than those of the other regions. Meanwhile, there were differences in baseline data among the five regions, including mean age, nationality, diet habit, smoking, and *Hp* infection rate, among others (**Table 3**).

**Table 3:** Differences of PG baseline levels among the different regions in the NAG group.

	Total (n = 1,590)	Southern China (n = 254)	Eastern China (n = 574)	Southwest China (n = 210)	Northeast China (n = 332)	Central/Northern China (n = 220)	P value
Sex							0.025
Male, n (%)	866	133 (52.4)^ab^	341 (59.4)^b^	117 (55.7)^ab^	164 (49.4)^a^	111 (50.5)^ab^	
Female, n (%)	724	121 (47.6)	233 (40.6)	93 (44.3)	168 (50.6)	109 (49.5)	
*Hp* infection							<0.001
Positive, n (%)	583	137 (53.9)^a^	235 (40.9)^b^	69 (32.9)^bc^	97 (29.2)^cd^	45 (20.5)^d^	
Negative, n (%)	1,007	117 (46.1)	339 (59.1)	141 (67.1)	235 (70.8)	175 (79.5)	
Nation							<0.001
Minority, n (%)	101	32 (12.6)^a^	0 (0)^b^	16 (7.6)^a^	33 (9.9)^a^	20 (9.1)^a^	
Han nationality, n (%)	1,489	222 (87.4)	574 (100)	194 (92.4)	299 (90.1)	200 (90.9)	
High-salt diet							<0.001
Yes, n (%)	240	41 (16.1)^a^	30 (5.2)^b^	37 (17.6)^a^	108 (32.5)^c^	24 (10.9)^a^	
No, n (%)	1,350	213 (84.9)	544 (94.8)	173 (82.4)	224 (67.5)	196 (89.1)	
Fruits							<0.001
Regular	1,005	101 (39.8)^a^	524 (91.3)^b^	122 (58.1)^c^	189 (56.9)^c^	124 (56.4)^c^	
Occasional	585	153 (60.2)	50 (8.7)	88 (41.9)	143 (43.1)	96 (43.6)	
Vegetables							<0.001
Regular	1,416	208 (81.9)^a^	547 (95.3)^b^	184 (87.6)^a^	291 (87.6)^a^	186 (84.5)^a^	
Occasional	174	46 (18.1)	27 (4.7)	26 (12.4)	41 (12.3)	34 (15.5)	
Milk							<0.001
Regular	373	19 (7.5)^a^	179 (31.2)^b^	90 (42.9)^c^	91 (27.4)^b^	57 (25.9)^b^	
Occasional	1,217	235 (92.5)	395 (68.8)	120 (57.1)	241 (72.6)	163 (74.1)	
Smoking							<0.001
Yes	410	80 (31.40)^a^	144 (25.1)^b^	53 (25.2)^b^	39 (11.7)^c^	94 (42.7)^a^	
No	1,180	172 (68.5)	430 (74.9)	157 (74.8)	293 (88.3)	126 (57.3)	
Age (years), mean (SD)	51.9 (9.9)	52.9 (8.6)^a^	49.1 (9.8)^b^	49.3 (9.3)^b^	55.9 (8.5)^c^	54.6 (11.2)^ac^	
PGI (ng/ml)	90.4 (85.2)	151.5 (124.1)^a^	63.8 (49.4)^b^	72.0 (49.2)^c^	119.7 (99.8)^d^	89.2 (82.1)^e^	
PGII (ng/ml)	8.3(11.7)	8.9 (9.8)^a^	7.4 (6.6)^b^	6.0 (5.1)^c^	11.0 (13.5)^d^	10.0 (8.0)^ae^	
PGR	10.1(16.0)	16.9 (13.4)^a^	7.9 (5.2)^b^	11.6 (7.9)^c^	11.5 (7.1)^dc^	8.0 (14.0)^be^	

^a,b,c,d,^ and ^e^: P > 0.05 when groups share the same letter.

P value refers to comparison among the five regions.

PGI, pepsinogen I; PGII, pepsinogen II; PGR, PGI/PGII ratio.

### Diagnostic Value of PG for Atrophy

The MAG and SAG groups were combined into the atrophy group, which was compared with the NAG group. The best cutoff for atrophy was estimated at PGI ≤73.1 ng/ml (AUC = 0.596) and PGR ≤9.8 (AUC = 0.636). When the *Hp* status was taken into consideration, the best cutoffs among the *Hp*-negative subgroup for atrophy were PGI ≤62.5 ng/ml (AUC = 0.623) and PGR ≤11.5 (AUC = 0.621). In the *Hp*-positive subgroup, the best cutoffs were PGI ≤90.2 ng/ml (AUC = 0.598) and PGR ≤8.8 (AUC = 0.627) (**Table 4**). The best cutoff values for atrophy in different regions were different (**Supplementary Table 1**).

**Table 4:** Diagnostic value of PG for atrophy, severe atrophy, and gastric cancer.

		Cutoff value	Sensitivity (95% CI), %	Specificity (95% CI), %	AUC (95% CI)	PPV%	NPV%	P
**Atrophy**								
		PGI ≤73.1 ng/ml	55.7 (50.2–61.1)	62.1 (59.7–64.5)	0.596 (0.573–0.618)	23.5	87.0	<0.0001
		PGR ≤9.8	71.1 (65.9–75.9)	51.8 (49.3–54.2)	0.636 (0.614–0.657)	18.3	91.4	<0.0001
	***Hp*−**	PGI ≤62.5 ng/ml	57.0 (48.9–64.8)	67.1 (64.1–70.0)	0.623 (0.594–0.651)	21.4	90.8	<0.0001
		PGR ≤11.5	79.1 (71.9–85.2)	46.1 (43.0–49.2)	0.621 (0.592–0.649)	3.6	99.1	<0.0001
	***Hp*+**	PGI ≤90.2 ng/ml	59.2 (51.5–66.6)	59.7 (55.6–63.7)	0.598 (0.562–0.633)	30.5	83.1	0.0001
		PGR ≤8.8	71.3 (63.9–77.9)	50.1 (45.9–54.2)	0.627 (0.592–0.662)	29.9	85.4	<0.0001
**Severe atrophy**								
		PGI ≤63.9 ng/ml	59.3 (45.7–71.9)	67.2 (65.0–69.3)	0.629 (0.607–0.651)	5.4	98.1	0.0004
		PGR ≤9.09	88.1 (77.1–95.1)	53.0 (50.7–55.3)	0.737 (0.717–0.757)	5.6	99.3	<0.0001
	***Hp*−**	PGI ≤62.5 ng/ml	68.2 (45.1–86.1)	64.5 (61.6–67.2)	0.616 (0.587–0.644)	18.7	93.3	0.0659
		PGR ≤9.1	77.3 (54.6–92.2)	57.8 (54.8–60.6)	0.644 (0.616–0.672)	3.4	99.2	0.0116
	***Hp*+**	PGI ≤75.4 ng/ml	64.9 (47.5–79.8)	64.3 (60.7–77.8)	0.669 (0.635–0.703)	8.5	97.3	0.0001
		PGR ≤4.5	56.8 (39.5–72.9)	86.3 (83.5–88.7)	0.768 (0.736–0.797)	16.1	97.7	<0.0001
**Gastric cancer**								
		PGI ≤43.5 ng/ml	40.0 (12.2–73.8)	86.5 (84.9–88.0)	0.616 (0.594–0.638)	1.5	99.6	0.2627
		PGR ≤4.7	60.0 (26.2–87.8)	89.0 (87.5–90.3)	0.715 (0.695–0.736)	2.7	99.8	0.0335
	***Hp*−**	PGI ≤57.5 ng/ml	80.0 (28.4–99.5)	68.7 (65.9–71.3)	0.697 (0.670–0.723)	0.8	99.7	0.2601
		PGR ≤7.1	80.0 (28.4–99.5)	77.6 (75.1–80.0)	0.797 (0.773–0.820)	1.5	99.9	0.0152
	***Hp*+**	PGI ≤89.7 ng/ml	60.0 (14.7–74.7)	55.9 (52.2–59.4)	0.575 (0.539–0.611)	0.9	99.5	0.5015
		PGR ≤4.3	60.0 (14.7–94.7)	86.4 (83.8–88.8)	0.640 (0.604–0.674)	2.8	99.7	0.4157

PGI, pepsinogen I; PGII, pepsinogen II; PGR, PGI/PGII ratio; Hp, Helicobacter pylori; CI, confident interval; AUC, area under the curve; PPV, positive predictive value; NPV, negative predictive value.

### Diagnostic Value of PG for Severe Atrophy

The NAG and MAG groups were combined into one group, which was compared with the SAG group. The best cutoff value for severe atrophy was PGR ≤9.09 (AUC = 0.737). In the *Hp*-positive subgroup, the best cutoff value was PGR ≤4.5 (AUC = 0.768) (**Table 4**). Because of the limited numbers of SAG cases in Central/Northern China and Southern China, those subgroups were not included in the analysis. The best cutoff for severe atrophy in Eastern China was PGR ≤4.5 (AUC = 0.680). The cutoff in Southwest China was PGR ≤7.4 (AUC = 0.813). The cutoff in Northeast China was PGR ≤8.9 (AUC = 0.781) (**Supplementary Table 1**).

### Diagnostic Value of PG for GC

The best cutoff for GC was PGR ≤4.7 (AUC = 0.715). When the *Hp* status was taken into consideration, the best cutoff in the *Hp*-negative subgroup for GC was PGR ≤7.1 (AUC = 0.797). There was no best cutoff in the *Hp*-positive subgroup (P > 0.05) (**Table 4**).

## Discussion

PGs can be used for GC screening, but the cutoffs vary among studies, and PG levels are influenced by numerous factors. The aim of the present study was to examine the diagnostic value of PG levels and *Helicobacter pylori* (*Hp*) status for screening GC and AG in asymptomatic individuals undergoing health checkup in China. This multicenter cross-sectional study suggested that serum PG levels can be used for screening GC and AG. The results suggest that different cutoff values should possibly be used in different *Hp* status groups and geographical regions, but it will have to be validated in the future using large-sample studies.

AG is an important turning point in the Correa theory (9). Here, in asymptomatic individuals undergoing health checkup, 14.5 and 17.3% participants showed AG and intestinal metaplasia, respectively, while 2.5% had severe AG (OLGA grades III and IV). Such patients have a high risk of developing GC within 5 years. Effective monitoring and intervention in this group are important measures to reduce the incidence of GC and improve the detection rate of early GC. Many factors were independently associated with AG in this work, among which high intake of fruits and vegetables may have a protective effect. Such benefit may be related to vitamin C levels, which are thought to reduce the formation of carcinogenic N-nitroso compounds in the stomach (31, 32). Meanwhile, previous studies have found a strong correlation between citrus fruits and gastric cancer (33). Therefore, the types of vegetables and fruits that could help prevent GC should be assessed in follow-up studies.

A meta-analysis of 31 studies involving a total of 1,520 patients with GC and 2,265 with AG found that serum PG levels have great potential as a non-invasive, population-based screening tool for GC and AG (17). The present study also confirmed that PGI levels and PGRs significantly decreased with the aggravation of atrophy, and PGR was even more significant. Impeding its wide application, PG levels are affected by race, region, age, gender, height, weight, body surface area, smoking, and alcohol consumption, among others (25). The cutoff values of PG for the diagnosis of atrophy vary among studies. Serum PGI ≤70 ng/ml and PGR ≤3 have been the most widely accepted values for detecting AG, with a sensitivity of 66.7–84.6% and a specificity of 73.5–87.1% (34–36). According to a European report, the cutoff values for fundus atrophy are PGI ≤56 ng/ml (sensitivity of 61.9% and specificity of 94.8%) and PGR ≤5 (sensitivity of 75.0% and specificity of 91.0%) (19). A study in Korea suggested that PGI ≤70 ng/ml has a good sensitivity (72.4%) for AG, but a low specificity (20.2%). The sensitivity and specificity of PGR ≤3 were found to be 59.2–61.7% and 61.0%, respectively (18). In the present study, the cutoff values for severe atrophy were significantly different from the above data in the *Hp*-positive subgroup (PGR ≤9.1 and PGR ≤4.5). In terms of the diagnostic value for GC, the cutoff value was PGR ≤4.7, but in the *Hp*-negative subgroup, the cutoff was PGR ≤7.1. Still, the number of patients with GC was small in the present study since the study population was made of healthy individuals receiving routine physical examination, and the PG cutoff values for GC were purely exploratory.

*Hp* infection has a significant effect on serum PG levels. In the present study PGI and PGII levels in the *Hp*-positive subgroup were higher than those of the *Hp*-negative subgroup, with PGII being higher and PGR being lower. In addition, *Hp* infection has been shown to participate significantly in the progression of gastric mucosal inflammation and the development of IM and AG (37, 38). As shown above, *Hp* prevalence was predominantly elevated in the MAG (49.8%) and SAG (65.3%) groups. China is a country with a high incidence of *Hp* infection, whose rate in the Chinese population is around 40–55% (20, 39–41). The overall *Hp* infection rate in the present study was 41.7%, similar to the literature (20). Therefore, using different cutoff values of PG for the *Hp*-negative and *Hp*-positive subgroups for detecting GC and AG could be considered, as well as in various regions with distinct *Hp* infection prevalence rates. Additional studies are required to determine the exact cutoff points of PG in different populations.

In the present study, PG levels in the NAG group showed significant differences among different regions of China. From the perspective of PGR, there were no significant differences between Eastern and Central/Northern China, but these levels were significantly different in Northeast and Southwest China. However, as shown in **Table 3**, there were differences in baseline data among regions, especially in *Hp* infection rate. Thus, differences in PG baseline levels among various regions may be related to these baseline data. Interestingly, there were significant differences in gender, nation, age, diet habit, smoking, and *Hp* infection rate between Eastern and Central/Northern China, but there was no difference in PGR levels. However, there was a significant difference in PGR levels between Eastern and Southwest China, with no differences in gender, smoking, age, and *Hp* infection rate, except for diet habit and nation. This suggests that in the subsequent studies assessing PG cutoff in China, it may be necessary to further consider the influence of ethnic groups (Southern China, Northeast China, and Southwest China are the places where ethnic minorities are gathered), dietary habits, *Hp* infection, and other factors.

This study had limitations. The numbers of patients with lesions and GC were small, requiring further sample size expansion to confirm the above results, especially in asymptomatic individuals. In addition, selection bias might exist since the included patients were individuals intending to undergo gastroscopy, suggesting good socioeconomic status. Large-scale and well-designed prospective studies are warranted for further validation of the cutoff values of PG in different regions in the *Hp*-negative and *Hp*-positive subgroups, combining lifestyle indexes such as diet habit and smoking in order to accurately determine the low-risk and high-risk groups for the screening of GC and AG.

In conclusion, dietary habits, smoking, age, and *Hp* infection are risk factors for GC and AG. Serum PG levels might be used for the screening of AG and GC. The results suggest that different cutoff values should possibly be used in different *Hp* status groups and geographical regions, but it will have to be validated in future studies with a large sample size. Future studies should also examine the value of PG levels for GC detection.

## Data Availability Statement

The datasets presented in this study can be found in online repositories. The names of the repository/repositories and accession number(s) can be found in the article/**Supplementary Material**.

## Ethics Statement

The studies involving human participants were reviewed and approved by the ethics committees of various participating hospitals. The patients/participants provided their written informed consent to participate in this study.

## Author Contributions

YT, YZ, and ZYS carried out the studies, performed the statistical analysis, and drafted the manuscript. HGW, XQH, HWX, HL, PS, LRG, HBW, HZX, YHL, DW, and SZL participated in acquisition, analysis, or interpretation of data. All authors contributed to the article and approved the submitted version.

## Funding

The work of YT was supported by the National Natural Science Foundation of China (#71804161) and the Zhejiang Province Major Disease Diagnosis and Treatment Technology Research Center (#17-2018-%s-0077). The work of ZS was supported by the China Foundation for Health Promotion (#CHPF-2015-WASC-001).

## Conflict of Interest

The authors declare that the research was conducted in the absence of any commercial or financial relationships that could be construed as a potential conflict of interest.

## Publisher’s Note

All claims expressed in this article are solely those of the authors and do not necessarily represent those of their affiliated organizations, or those of the publisher, the editors and the reviewers. Any product that may be evaluated in this article, or claim that may be made by its manufacturer, is not guaranteed or endorsed by the publisher.
