# Supplementary material for: Diagnostic Value of Serum Pepsinogen Levels for Screening Gastric Cancer and Atrophic Gastritis in Asymptomatic Individuals: A Cross-Sectional Study
Source: Front Oncol. 2021 Aug 24;11:652574. doi: 10.3389/fonc.2021.652574 (PMC8421685; doi:10.3389/fonc.2021.652574)
Supplement: Supplementary file 1 [file Table_1.docx]

**Supplementary table 1**

Diagnostic value of PG for atrophy, severe atrophy in different regions of China.

|  |  | Cutoff value | | Sensitivity (95%CI), % | Specificity (95%CI), % | AUC (95%CI) | PPV% | NPV% | P |
| --- | --- | --- | --- | --- | --- | --- | --- | --- | --- |
| **Atrophy** |  |  | |  |  |  |  |  |  |
|  | **Southern China** | PGI ≤280.2 ng/ml | | 66.7 (9.4-99.2) | 84.3 (79.2-88.5) | 0.588 (0.525-0.649) | 0.5 | 95.3 | 0.7508 |
|  |  | PGR ≤18.9 | | 100 (29.2-100) | 41.1 (35.0-47.4) | 0.611 (0.549-0.671) | 1.3 | 99.0 | 0.4874 |
|  | **Eastern China** | PGI ≤60.9 ng/ml | | 61.1 (54.0-67.8) | 54.2 (50.0-58.3) | 0.579 (0.544-0.614) | 32.3 | 79.1 | 0.0006 |
|  |  | PGR ≤9.0 | | 77.8 (71.5-83.3) | 38.5 (34.5-42.6) | 0.606 (0.570-0.640) | 31.4 | 82.6 | <0.0001 |
|  | **Southwest China** | PGI ≤104.7 ng/ml | | 44.6 (31.3-58.5) | 78.0 (71.8-83.4) | 0.576 (0.514-0.636) | 16.8 | 64.3 | 0.0896 |
|  |  | PGR ≤12.5 | | 80.4 (67.6-89.8) | 44.5 (37.6-511.5) | 0.648 (0.587-0.705) | 28.4 | 88.5 | 0.0002 |
|  | **Northeast China** | PGI ≤120.6 ng/ml | | 69.2 (54.9-81.3) | 51.2 (45.7-56.7) | 0.594 (0.543-0.644) | 8.6 | 89.0 | 0.0190 |
|  |  | PGR ≤11.5 | | 65.4 (50.9-78.0) | 49.7 (44.2-55.2) | 0.555 (0.503-0.605) | 17.2 | 95.1 | 0.1981 |
|  | **Central/Northern China** | PGI ≤113.0 ng/ml | | 87.5 (47.3-99.7) | 64.6 (57.8-70.9) | 0.745 (0.683-0.800) | 2.0 | 91.8 | 0.0001 |
|  |  | PGR ≤9.3 | | 75.0 (34.9-96.8) | 57.7 (50.9-64.3) | 0.631 (0.565-0.694) | 3.1 | 94.0 | 0.2237 |
| **Severe atrophy** | | |  |  |  |  |  |  |  |
|  | **Eastern China** | PGI ≤63.9 ng/ml | | 73.7 (56.9-86.6) | 47.4 (43.7-51.0) | 0.565 (0.529-0.600) | 7.0 | 96.4 | 0.1354 |
|  |  | PGR ≤4.5 | | 42.1 (26.3-59.2) | 90.1 (87.7-92.2) | 0.680 (0.646-0.713) | 18.2 | 96.3 | 0.0002 |
|  | **Southwest China** | PGI ≤117.3 ng/ml | | 66.7 (22.3-95.7) | 80.7 (75.4-85.3) | 0.701 (0.642-0.755) | 1.7 | 92.6 | 0.1003 |
|  |  | PGR ≤7.4 | | 83.3 (35.9-99.6) | 77.6 (72.0-82.5) | 0.813 (0.761-0.858) | 9.4 | 99.0 | <0.0001 |
|  | **Northeast China** | PGI ≤71.9 ng/ml | | 40.0 (5.3-85.3) | 88.9 (85.3-91.9) | 0.503 (0.452-0.555) | 4.5 | 98.5 | 0.9840 |
|  |  | PGR ≤8.9 | | 100 (47.8-100) | 67.8 (62.8-72.5) | 0.781 (0.737-0.822) | 4.6 | 99.6 | <0.0001 |
